# Evaluation of BIRC6 Expression in Oral Squamous Cell Carcinoma, Epithelial Dysplasia, Lichen Planus with and without Dysplasia, and Hyperkeratosis

**DOI:** 10.3390/diagnostics13233560

**Published:** 2023-11-29

**Authors:** Fateme Eskandari, Alireza Razavian, Razieh Zare, Shayan Ejlali, Alireza Razmahang, Milad Zanjani, Seyedeh Sara Aghili, Mohammad Amin Mahdiyar, Hossein Mofidi, Kamyar Abbasi, Ashkan Badkoobeh, Nafiseh Shamloo, Lotfollah Kamali Hakim, Ahmed Hussain, Hamid Tebyaniyan

**Affiliations:** 1 School of Dentistry, Shiraz University of Medical Sciences, Shiraz 71348-14336, Iran; fateme.eskandarii95@gmail.com (F.E.); saraaghili95@gmail.com (S.S.A.); 2 Department of Endodontics, Semnan Dental School, Semnan University of Medical Sciences, Semnan 35147-99442, Iran; 3 Oral and Maxillofacial Research Center, Department of Oral and Maxillofacial Pathology, School of Dentistry, Shiraz University of Medical Sciences, Shiraz 71348-14336, Iran; 4 School of Dentistry, Borujerd Branch, Islamic Azad University, Borujerd 69151-36335, Iran; shayan.ejlali72@gmail.com; 5 Molecular Pathology Research Center, Shiraz University of Medical Sciences, Shiraz 71348-14336, Iran; razmahang.alirezaa@gmail.com; 6 Department of Oral and Maxillofacial Surgery, School of Dentistry, Shiraz University of Medical Sciences, Shiraz 71348-14336, Iran; milad_zanjani@yahoo.com; 7 Department of Orthopedics, School of Medicine, Shiraz University of Medical Sciences, Shiraz 71348-14336, Iran; 8 Department of Endodontics, School of Dentistry, Shiraz University of Medical Sciences, Shiraz 71348-14336, Iran; 9 Department of Prosthodontics, School of Dentistry, Shahid Beheshti University of Medical Sciences, Tehran 19839-69411, Iran; 10 Department of Oral and Maxillofacial Surgery, School of Dentistry, Qom University of Medical Sciences, Qom 37136-49373, Iran; 11 Department of Oral and Maxillofacial Pathology, School of Dentistry, Shahid Beheshti University of Medical Sciences, Tehran 19839-69411, Iran; 12 Independent Researcher, Tehran 16738-48110, Iran; arman_kamali.hakim@yahoo.com; 13 School of Dentistry, Edmonton Clinic Health Academy, University of Alberta, Edmonton, AB T6G 1C9, Canada; 14 Department of Science and Research, Islimic Azade University, Tehran 15847-15414, Iran

**Keywords:** BIRC6, epithelial dysplasia, lichen planus, malignant transformation, oral squamous cell carcinoma

## Abstract

Background: BIRC6, regarded as the pivotal member of the inhibitor of the apoptosis (IAP) family, has been linked to the development of different types of cancer in humans. The objective of this study was to examine the expression of BIRC6 in various oral conditions, including OLP with dysplasia (OLPD), hyperkeratosis (HK), OLP, epithelial dysplasia (ED), and oral squamous cell carcinoma (OSCC), to investigate its potential involvement in the development of OSCC and the pathogenesis and malignant transformation of OLP, which is known as a precancerous condition. Methods: In this retrospective cross-sectional study, 99 cases, consisting of 19 cases of OSCC, 21 cases of ED, 23 cases of OLP, 20 cases of OLPD, and 16 cases of HK as the control group, were investigated regarding BIRC6 expression by immunohistochemical staining. After that, the immunohistochemical expression of BIRC6 in the epithelial compartment was analyzed. Statistical analysis was performed to investigate the relationship between the expression of BIRC6 and clinicopathological variables. The statistical analysis of the data involved the use of one-way ANOVA, post hoc Tukey, Kruskal–Wallis, Chi-square, Spearman’s correlation, and Mann–Whitney tests. The significance level was set at *p* < 0.05. Results: Positive BIRC6 staining was found in 91.7% of the subjects of OLP, 88.1% of HK, 86.1% of ED, 93% of OLPD, and 94.7% of OSCC. OSCC showed the highest BIRC6 expression (*p* = 0.00). The average total staining score was remarkably greater in OSCC and dysplastic lesions compared with HK (*p* = 0.00, *p* = 0.00). Conclusions: While the current study suggested that BIRC6 may play a role in the tumorigenesis of OSCC, its role in the malignant transformation of OLP has yet to be definitively established.

## 1. Introduction

Oral health plays a significant role in maintaining general health [1,2]. Oral squamous cell carcinoma (OSCC) is the prevailing malignant tumor among all types of squamous cell carcinomas that occur in the head and neck region [3], ranking 18th globally in 2018 [4,5]. OSCC is known for its propensity to recur, metastasize, and invade local tissues [6]. To effectively manage this condition, a combination of treatment modalities such as surgery, radiation, and/or chemotherapy is often required [7]. Despite significant advancements in the treatment of OSCC, the survival rate for this disease remains poor [8,9,10]. These challenges highlight the need for continued research into new treatment strategies and biomarkers for early detection and improved patient outcomes [11].

Oral lichen planus (OLP) stands among the chronic inflammatory diseases of the oral mucosa [12,13]. The possibility of malignant transformation in OLP remains controversial, and its rate is estimated to be between 0.5% and 12%, with the mechanism of transformation still unknown [14,15].

Dysplasia refers to abnormal growth, and the grade of oral epithelial dysplasia (ED) helps assess the likelihood of a lesion becoming malignant. The presence of oral epithelial dysplasia is considered the gold standard for managing oral potentially malignant disorders [16,17]. The inhibitor of the apoptosis protein (IAP) family has been shown to play a crucial role in the apoptosis of cancerous cells. IAPs are characterized by 1–3 copies of the Baculoviral IAP Repeat [3] domain and can inhibit apoptosis in cancerous cells by binding and suppressing proapoptotic factors. The most prominent member of the IAP family is BIRC6, also known as Apollon or Brcue [18,19]. Compared with other IAPs, BIRC6 has been shown to play a cytoprotective role [20,21] and is also involved in regulating cytokinesis, the final event in cell division [20]. These dual functions of BIRC6 in both cell death and division make it a promising target for targeted therapy in various types of cancer. Notably, the BIRC6 protein can bind to active caspase-3, -6, -7, and -9, inhibiting the caspase cascade and ultimately leading to apoptosis [22].

BIRC6 expression has been suggested by many researchers to be correlated with tumor stage and differentiation, angiogenesis, chemotherapeutic response, and other clinical properties in the context of cancer [23]. Previous studies have indicated that the prognostic value of the BIRC6 protein is highly cancer-specific [24,25]. Furthermore, evidence demonstrates the overexpression of BIRC6 in various types of cancer, including colorectal cancer, brain cancer, childhood de novo acute myeloid leukemia, hepatocellular carcinoma, and human epithelial ovarian cancer [18,26,27,28]. However, low BIRC6 expression (0.0%) in salivary gland adenoid cystic carcinoma suggests a dual function for BIRC6 in carcinogenesis [29]. To our knowledge, this study is the first to investigate the expression of BIRC6 in oral lichen planus with dysplasia (OLPD), hyperkeratosis (HK), OLP, ED, and OSCC. This study aimed to evaluate the role of BIRC6 in patients with OLPD, HK, OLP, ED, and OSCC and to shed light on the potential mechanisms underlying oral carcinogenesis.

## 2. Materials and Methods

### 2.1. Patients and Tissue Selection

In this cross-sectional study, 99 histological samples were selected from the archive of the Oral and Maxillofacial Pathology Department (affiliated to Shiraz University of Medical Sciences, School of Dentistry) between 2011 and 2018, among which, 19 cases were diagnosed with OSCC, 21 cases with ED, 23 patients with OLP, 20 cases with OLPD, and finally 16 cases with HK. Subsequently, the hematoxylin-eosin (H&E) slides of the selected pathological lesions were re-evaluated to confirm the result of the previous diagnosis. In this study, specimens lacking a definitive diagnosis, displaying systemic diseases, featuring tumors in other body sites, or lacking adequate epithelial components were excluded from consideration. Erosive and ulcerative OLPs were also excluded. We gathered patients’ demographic information and clinical details, including age, gender, and lesion location, from their medical records. Subsequently, we conducted histopathologic grading for dysplastic and tumoral samples, collecting the resulting data for future reference.

### 2.2. Ethical Approval

This study design was approved by the Ethics in Human Research Committee of Shiraz University of Medical Sciences (IR.SUMS.DENTAL.REC.1398.135). It was performed in full accordance with ethical principles, including the World Medical Association Declaration of Helsinki (version 2008). Informed consent was obtained from all participants in the present study.

### 2.3. Immunohistochemistry

To perform immunohistochemical evaluations, sections of sample tissue were first prepared using a microtome with a thickness of 4 µm, and then the prepared tissue sections were mounted and fixed on glass slides. Paraffin-embedded sections of all the samples were deparaffinized in xylene and rehydrated in a decreasing serial dilution of ethanol in distilled water. Routine H&E staining was conducted on the rehydrated sections for histopathologic analysis. Further analyses were performed using immunohistochemistry (IHC) staining by labeling the sections with immunohistochemistry streptavidin–biotin and subsequent incubation with polyclonal anti-BIRC6 antibody (ab19609, Abcam company, Germany). A brown-stained cytoplasm was considered positive for the staining, and a sample with no primary antibody coating was considered negative. Of note, healthy brain tissue was used as a positive control. Typically, the stained samples were evaluated using a light microscope with ×400 magnification for each slide, through which the amount of positively stained area was utilized to allocate intensity and percentage of BIRC6 gene expression. We observed more stained areas in the slides with heterogenous staining. The percentage of the positive cells was assessed in 1000 cells of each lesion at ×400 magnification. The intensity of immunohistochemical staining was also scored from 1 to 3 (1, weak; 2, intermediate; and 3, strong), and the number of the positively stained cells was scaled from 0 to 3 (0, 0% positive cells; 1, 1–10% positive cells; 2, 11–50% positive cells; and 3, >50% positive cells) by two evaluators. The final score of each sample was achieved by multiplying the scores of staining intensity and percentage of positive cells, which was then classified as a low expression of BIRC6 when the final scores were between 1 and 3 and high when they were from 4 to 9 [30]. 

### 2.4. Statistical Analysis

Using the SPSS statistical software (version 18.0), the data were statistically analyzed using one-way ANOVA, post hoc Tukey test, and Kruskal–Wallis tests to compare the mean values of BIRC6 expression in the study’s groups. The intensity of staining and the final scores of each specimen were further analyzed using the Chi-square test. Spearman’s correlation and Mann–Whitney tests were also utilized to compare BIRC6 expression with demographic variables such as age and gender, respectively. *p*-values less than 0.05 were considered significant.

## 3. Results

In this study, 99 participants were included, consisting of 46 men and 53 women, with a mean age of 50.8 years old (min = 15, max = 83). An effective monitoring and evaluation required collecting baseline data of all the study samples, summarized in Table 1. No significant association was detected among age, gender (*p* = 0.422, *p* = 0.854), or other clinicopathologic characteristics of the patients.

**Figure 1:**
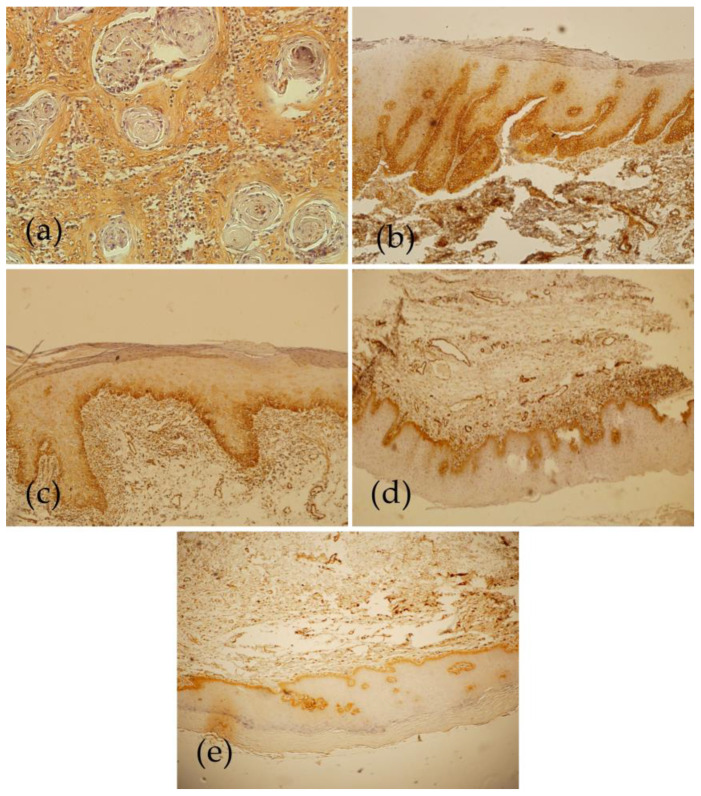
(**a**) Immunohistochemical staining of BIRC6 in OSCC: peripheral cells staining but no staining in central cells and keratin pearls (×400). (**b**) Immunohistochemical staining of BIRC6 in ED: positive staining in the basal and suprabasal layers (×400). (**c**) Immunohistochemical staining of BIRC6 in OLPD: positive staining in the basal and suprabasal layers (×400). (**d**) Immunohistochemical staining of BIRC6 in the basal and suprabasal layers of OLP (×400). (**e**) Immunohistochemical staining of BIRC6 in the basal layer of HK (×400).

**Figure 2:**
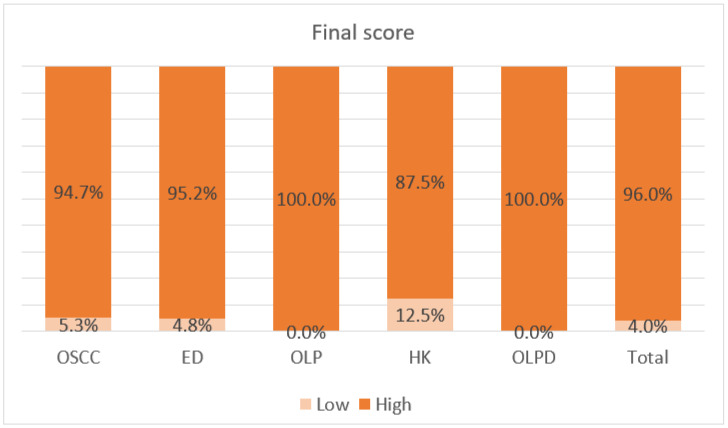
BIRC6 mean final score in all study groups.

Positive cytoplasmic BIRC6 expression, indicated by a brown stain, was observed in epithelial cell components in all the study samples. In this regard, Table 2 shows the overall staining intensity and BIRC6 expression percentage in the epithelial components of the samples. In the stroma, the staining was limited to the endothelial cells and inflammatory cells, while fibroblast cells of the stroma showed no positive staining. Notably, OSCC had the highest central layer mean percentage of BIRC6 expression (94.73%) among the defined groups. Of these positively stained OSCC, 94.7% showed central layer staining, and all (100%) finally showed staining in the epithelial cells (Figure 1). Peripheral cell layers indicated higher staining percentage and intensity than the central layers (Table 2). The final scores are demonstrated in Figure 2; in total, 94.7% of the subjects had high scores, and only a few cases (5.3%) obtained low scores. Notably, individual cell keratinization and cells around the keratin pearls and central area of large sheets were not positively stained, but small cords and nests were entirely stained.

In the samples categorized in the ED group, positive BIRC6 staining was observed, and the mean percentage of BIRC6 expression attained in the suprabasal and basal layers was 86.19% and 99.04%, respectively (Figure 1). Generally, in the final scoring, BIRC6 expression was defined as high in 95.2% of the ED samples and as low in very few cases (4.8%) (Figure 1). Of note, the superficial keratins were not stained.

The second highest value for BIRC6 expression was observed from the OLPD samples, with a mean percentage of 96.5% (Figure 1). The overall staining intensity of the suprabasal layer was 1+, which was strong in the basal layer (Table 2). Finally, our analysis determined that all the OLPD samples (100%) produced a high score for BIRC6 expression (Figure 1).

In the OLP specimens, the mean value of BIRC6 expression was 91.7% in the suprabasal layer and 100% in the basal layer (Figure 1). In most cases, staining intensity was moderate in the suprabasal layer and strong in the basal layer (Table 2). Also, BIRC6 expression was high in all the samples (Figure 1). The inflammatory cells were stained near the basal layer.

In the HK group, the suprabasal layer had 88.12% BIRC6 expression (Figure 1). Only two cases (9.5%) from the HK samples showed a low expression of BIRC6, and the rest of them (90.5%) exhibited a high expression (Figure 1).

As mentioned earlier, the highest mean BIRC6 expression in the central layer was seen in the OSCC group. In all the samples, staining was more evident in basal/peripheral cells compared with suprabasal/central layers.

**Figure 3:**
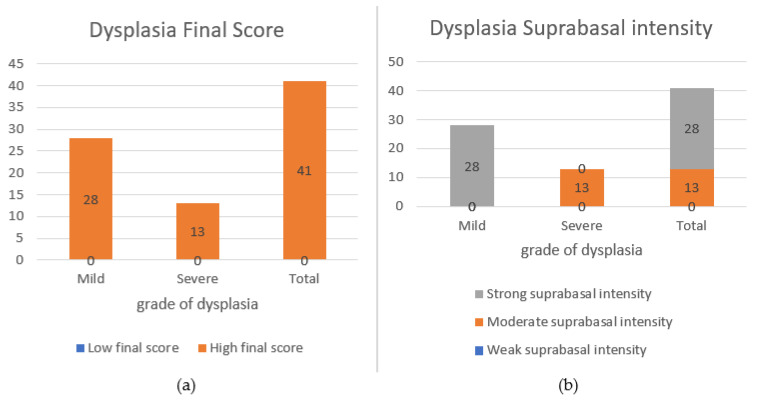
(**a**) Mean final score in different grades of dysplasia groups and (**b**) intensity in different grades of dysplasia groups.

The degree of BIRC6 expression in basal and suprabasal layers among different groups was evaluated using the Kruskal–Wallis test. The data elucidated that the mean BIRC6 expression in the suprabasal layer significantly differed among groups (*p* < 0.05). Moreover, the staining intensity and the final score of BIRC6 expression in the suprabasal layer differed significantly among these groups (*p* < 0.05). In the light of analysis via Dunn test, it was found that the suprabasal percentage in OSCC was higher than that of the HK and ED groups (*p* = 0.012). The intensity of suprabasal staining and the final score also appeared to be significantly different among the groups, which were remarkably greater in the samples of OSCC compared with the achieved values in HK (*p* = 0.01, *p* = 0.01). In addition, they were higher in the ED groups (ED and OLPD) than in the HK group (*p* = 0.01). The final scores in different grades of the dysplasia group are listed in Figure 3. The Mann–Whitney test was used to evaluate the relationship between the grade of dysplasia and BIRC6 expression, which showed a significant difference among suprabasal intensity, suprabasal final score, and the grade of dysplasia (*p* < 0.001, *p* < 0.001). In other words, as the intensity and final score of BIRC6 expression in the suprabasal layer elevated, the grade of dysplasia significantly increased (Figure 3). More precise stratification showed no significant difference between other clinicopathologic factors, such as the grade of OSCC, the percentage of BIRC6 expression, staining intensity, and the final score (*p* > 0.05). Utilization of the Chi-square test and one-way ANOVA highlighted no difference between the clinicopathologic manifestations and the two variables of gender and age (*p* > 0.05). 

## 4. Discussion

The modified expression of BIRC6 protein has been implicated in the pathogenesis of several human malignancies, making its expression a critical factor to study. BIRC6, also known as Apollon or Bruce, is a crucial member of the IAP family that performs its function by binding to caspases-3, -6, -7, -9, and SMAC and inhibiting their activities through its BIR domain [31]. In this study, elevated BIRC6 expression was observed more frequently in OSCC, LPD, and ED compared with in HK. Additionally, BIRC6 expression was more intense in neoplastic tissue than in dysplastic or nondysplastic tissues, suggesting a potential role for BIRC6 overexpression in the carcinogenesis of OSCC. These results are consistent with previous studies by Dong et al. and Gharabaghi et al. [27,32].

The upregulation and overexpression of BIRC6 have been detected in various types of tumors, including gastric carcinoma, colorectal cancer, and primary breast cancer, compared with normal tissues [18,21,33]. However, BIRC6 has also been identified as a crucial regulator of cytokinesis due to its vital role in cell proliferation [25,34]. These diverse roles in different tumors may be due to the differential activation of different domains of BIRC6, as well as various molecular interactions with BIRC6. For example, BIRC6 can facilitate the proteasomal degradation of proapoptotic protein caspase-9 through its UBC domain. Additionally, through its BIR domain, it can bind to active caspases and inhibit them, including caspase-3, -6, -7, and -9 [18]. Thus, variations in BIRC6 play a role in tumorigenesis that may be related to diverse genetic defects that affect BIRC6 expression in unique tumor types [20]. The scoring method and type of antibodies used have also been found to influence the results of studies [21]. Therefore, in this study, quantitative, semiquantitative, and staining intensity methods were used to compare the study samples, and all variables confirmed the results.

Our study found that BIRC6 was overexpressed in OSCC cases, and to the best of our knowledge, this is the first report to clarify BIRC6 expression in OSCC. However, Banakar et al. assessed the serum level of BIRC6 in OSCC and found no significant difference in the serum levels of BIRC6 between patients with OSCC and healthy individuals [35]. Other studies have also investigated antiapoptotic markers in OSCC. Mallick et al. reported that the expression of Mcl-1, which inhibits apoptosis similarly to BIRC6, was significantly increased in OSCC compared with normal mucosa [36]. Another study detected alterations in proteins associated with apoptosis in malignant lesions of the oral epithelium [37]. Poomsawat et al. evaluated the expression of Survivin, another antiapoptotic protein participating in the regulation of cytokinesis similarly to BIRC6, and found that Survivin expression was elevated in OSCC. They suggested this antiapoptotic protein increases cell proliferation [34,38]. Additionally, S. X. Li et al. conducted a study on the expression of caspase-3 in OSCC. They observed that the expression of activated caspase-3 was not found in OSCC, and the expression of caspase-3 zymogen decreased in OSCC [39]. Interestingly, BIRC6 has also been found to play a role in cytokinesis by regulating the ubiquitination and degradation of Aurora B kinase [25,34]. This suggests that BIRC6 may have multiple functions in tumorigenesis beyond its role in apoptosis inhibition.

Studies have shown that BIRC6 can regulate the expression of cyclin D1, a vital regulator of the G1 phase of the cell cycle, in cancer cells [40]. BIRC6 has also been shown to interact with cyclin-dependent kinase 1 (CDK1), a protein that plays a critical role in regulating the cell cycle [40] and promoting its activity [18,40]. This suggests BIRC6 may promote cell proliferation by regulating cyclin expression and CDK activity. In addition, BIRC6 is positively correlated with Ki-67 expression in various types of cancer, including breast cancer, hepatocellular carcinoma, and colorectal cancer. This suggests that BIRC6 may also play a role in regulating cell proliferation by promoting Ki-67 expression. However, the exact mechanisms by which BIRC6 regulates cyclin and Ki-67 expression still need to be fully understood and further investigated [18].

As stated earlier, we detected that the average percentage of BIRC6 expression was significantly higher in OSCC than in HK cases, which was the control group. Similarly, Salehi et al. detected a higher BIRC6 expression in gastric carcinoma tissues compared with the normal margin of gastric tissues [21]. Gharabaghi et al. also reported that the expression of BIRC6 was remarkably higher in tumor tissues than in normal tissues [32]. So, BIRC6 can be suggested as a marker contributing to tumor surveillance and progression.

The elevated BIRC6 expression in the basal or peripheral cells of the specimens, especially the OSCC samples, agreed with the findings of previous studies in gastric carcinoma and childhood acute myeloid leukemia [20,21]. In the OSCC group, small nests, cords, and tumoral single cells indicated higher BIRC6 expression than large nests and sheets. Accordingly, these results may approve the higher expression of BIRC6 in tumor cells with a basal-like phenotype. Due to the small size of the studied OSCC samples, we could not investigate the relationship between the expression of BIRC6 and the mode of invasion in tumors. 

The expression and function of apoptotic proteins in OLP are still unknown [41,42]. Our findings demonstrated that the final score of BIRC6 expression in OLP was significantly lower than in OLPD and malignancies. Consistent with our results, Mattila et al. reported that caspase-3 was mainly located in the basal and parabasal cells. Also, caspase-3 was observed in the cytoplasm of the epithelial cells, which could consequently facilitate the activity of BIRC6 [22]. In OLP, apoptosis is inhibited by several antiapoptotic factors [43,44]. Tobon et al. indicated that the expression of caspase-3 was strong in basal epithelial cells and further suggested that caspase is active in the proliferating compartment of OLP lesions. Moreover, they detected a high expression of caspase-3 in OLP compared with the controls. Tobon et al. showed that keratinocyte apoptosis and caspase-3 expression near the basal and parabasal epithelial layers may be suggestive of the issue that proliferating epithelial cells may aim to be disrupted in OLP. They also suggested a role for caspase-3 in keratinocyte differentiation in both normal and diseased oral mucosa [45]. Hence, lower BIRC6 expression in OLP, rather than OLPD and OSCC, is related to the strong presence of caspase-3 in OLP, since BIRC6 binds to and inhibits this caspase [22,45]. According to LEITE’s data, the apoptotic area index (caspase-3) of actinic cheilitis without epithelial dysplasia was more than that of the samples with epithelial dysplasia [46].

We also observed that BIRC6 expression was significantly higher in the dysplastic groups than in HK. As we know, Bcl-2 typically blocks the activation of caspase-3. After that, when Bax blocks Bcl-2 activity, caspase-3 activity is unchecked, and apoptotic cell death proceeds. Similarly, S Juneja et al. studied Bcl-2, an oncoprotein restraining apoptosis-like BIRC6 in ED, and showed that along with increasing the grade of dysplasia, the expression of the Bcl-2 protein elevated [47]. These findings suggest the role of apoptosis in the development of ED. In addition, the failure of apoptosis may consequently result in the selective survival of genetically deregulated cells that could be known as potentially malignant disorders [48].

Consistent with our findings, Muzio et al. stated that a high expression of one of the IAP members, named Survivin, was detected in 97% of oral epithelial dysplasia samples. As BIRC6 and Survivin both play roles in suppressing caspases, including caspase-3, -7, and initiator caspase-9, we can attribute this relationship to caspase activity between our finding and that of Muzio et al.’s study [49,50]. Furthermore, LEITE et al. showed that oral leukoplakia cases were positive for cleaved caspase-3 due to etiological factors involved in premalignant lesion formation [46]. We should also consider the differences in apoptosis detection methods, analysis methods, and study models [51].

Some studies suggested that the number of apoptotic cells, such as caspase-3 and Survivin, decreases in oral lesions due to the increased molecular abnormalities in epithelial cells and tumors [46].

In the present study, no correlation was observed between different grades of tumor and BIRC6 expression. Some studies have shown that BIRC6 expression could be related to the grade of tumors, for instance, in non-small-cell lung carcinoma and prostate cancer [27,32,40]. Accordingly, this could result from employing a relatively limited number of samples. Therefore, further studies are needed to clarify the roles of BIRC6 in tumorigenesis, metastasis, and the grade of tumors.

In normal oral tissue, BI has rarely been described in humans, and the four reports available are similar to what we reported herein. We can describe BIRC6 expression with a broader range of functional alterations, and all benign cases were positive, which is not in line with Salehi et al. and Tang et al.’s studies [21,25].

The current study also detected BIRC6 expression in the cytoplasm of tumor cells, confirming previous studies showing that it is localized to the Golgi compartment and the vesicular system [52]. Similarly, Garrison et al. reported that BIRC6 staining is chiefly observed in the cytoplasm of a cell [33]. Additionally, Chen et al.’s study reported that the expression of the BIRC6 protein was observed in most cancer samples (81%) and determined cytoplasm as the location of BIRC6 protein expression [34]. 

Yoshihara et al. reported the idea of basket clinical trials, wherein patients are assigned experimental treatments according to their genomic profile instead of the tumor’s tissue of origin. Consequently, the results of the current study may pave the way for novel avenues in the diagnosis and management of tumors [53].

To the authors’ knowledge, this is the first study elucidating BIRC6 expression in OED, OLP, OLPD, and HK. These findings can be attributed to the known functions of the BIRC6 protein. However, further studies are needed to investigate different aspects of BIRC6 function in these lesions.

Overall, the present study provides important insights into the expression of BIRC6 in various oral lesions and suggests that BIRC6 may play a role in the tumorigenesis of OSCC. However, additional studies involving larger sample sizes are required to validate these results and gain a more comprehensive understanding of the underlying mechanisms through which BIRC6 contributes to the development of oral cancer.

One of the limitations of this current research is the limited number of cases that were studied. Therefore, further investigations with larger sample sizes are needed.

## 5. Conclusions

The present study found a significant increase in BIRC6 protein expression in OSCC, ED, OLP, and OLPD compared with HK. These findings suggest that BIRC6 may serve as a novel and effective marker for predicting and diagnosing these diseases, and the early detection of BIRC6 expression may improve patient outcomes. This study confirmed the role of BIRC6 in the carcinogenesis of OSCC, but its function in the malignant transformation of OLP was not established. Further investigations are needed to determine whether BIRC6 can be used as an independent prognostic factor to guide treatment and improve survival rates for these diseases. These findings provide important insights into the potential clinical utility of BIRC6 as a biomarker for oral lesions and highlight the need for further research to fully elucidate its role in oral carcinogenesis.

## Figures and Tables

**Table 1:** Baseline data of all study groups.

Variable		Total *n* = 99		OSCC *n* = 19		Epithelial Dysplasia *n* = 21		Oral Lichen Planus *n* = 23		Hyperkeratosis *n* = 16		Oral Lichen Planus Dysplasia *n* = 20		*p*-Value
Age		50.8	14.1	54.50	14.20	48	12.80	52.30	13.40	48.40	14.50	52	18.1	0.422
Gender	F	53	53.5%	10	18.9%	13	24.5%	13	24.5%	7	13.2%	10	18.9%	0.854
	M	46	46.5%	9	19.5%	8	17.4%	10	21.7%	9	19.5%	10	21.7%	
SCC grade	Well-differentiated	14	68.4%	13	100.0%	0	0.0%	0	0.0%	0	0.0%	0	0.0%	NA
	Moderate	5	31.6%	5	100.0%	0	0.0%	0	0.0%	0	0.0%	0	0.0%	
Dysplasia grade	Mild	25	60.9%	0	0.0%	5	20.0%	0	0.0%	0	0.0%	20	80.0%	NA
	Severe	16	39.1%	0	0.0%	16	100.0%	0	0.0%	0	0.0%	0	0.0%	

**Table 2:** BIRC6 overall intensity and percentage in all groups’ basal/suprabasal layer.

Group	Peripheral/Basal Intensity			Central/Suprabasal Intensity			Central/Suprabasal Percentage			Peripheral/Basal Percentage		
	Mean ± SD	Median		Mean ± SD	Median		Mean ± SD	Median		Mean ± SD	Median	
		Minimum	Maximum		Minimum	Maximum		Minimum	Maximum		Minimum	Maximum
OSCC	2.57 ± 0.60 A *	3		2.42 ± 0.60 A	2		94.73 ± 6.96 A	100		100 ± 0.00 A	100	
		1	3		1	3		100	100		100	100
ED	2.58 ± 0.58 A	3		2.00 ± 0.51 AB	2		89.58 ± 12.32 AB	90		99.16 ± 4.08 A	100	
		1	3		1	3		40	100		80	100
OLP	2.69 ± 0.54 A	3		1.96 ± 0.34 AB	2		100 ± 0.00 AB	100		89.23 ± 6.27 A	90	
		1	3		1	3		100	100		70	100
HK	2.42 ± 0.67 A	3		1.57 ± 0.59 B	2		86.19 ± 9.73 B	90		99.04 ± 4.36 A	100	
		1	3		1	3		50	100		80	100
OLPD	2.77 ± 0.44 A	3		1.88 ± 0.78 AB	2		91.11 ± 3.33 AB	90		100 ± 0.00 A	100	
		2	3		1	3		90	100		100	100
*p*-value	0.532			<0.001			0.006			0.655		

* Kruskal–Wallis H test. In each column, mean rank values with at least a common letter were not statically significant (Dunn’s post hoc test).

## Data Availability

The datasets used and/or analyzed during the current study are included in this article.

## References

[1] Hasanzade M., Zabandan D., Mosaddad S., Habibzadeh S. (2023). Comparison of marginal and internal adaptation of provisional polymethyl methacrylate restorations fabricated by two three-dimensional printers: An in vitro study. Dent. Res. J..

[2] Golfeshan F., Mosaddad S.A., Ghaderi F. (2021). The Effect of Toothpastes Containing Natural Ingredients Such as Theobromine and Caffeine on Enamel Microhardness: An In Vitro Study. Evid. Based Complement. Altern. Med..

[3] Habibzadeh S., Ghoncheh Z., Kabiri P., Mosaddad S.A. (2023). Diagnostic efficacy of cone-beam computed tomography for detection of vertical root fractures in endodontically treated teeth: A systematic review. BMC Med. Imaging.

[4] Aghili S.S., Zare R., Jahangirnia A. (2023). Evaluation of Paxillin Expression in Epithelial Dysplasia, Oral Squamous Cell Carcinoma, Lichen Planus with and without Dysplasia, and Hyperkeratosis: A Retrospective Cross-Sectional Study. Diagnostics.

[5] Mosaddad S.A., Namanloo R.A., Aghili S.S., Maskani P., Alam M., Abbasi K., Nouri F., Tahmasebi E., Yazdanian M., Tebyaniyan H. (2023). Photodynamic therapy in oral cancer: A review of clinical studies. Med. Oncol..

[6] Sufianov A., Begliarzade S., Kudriashov V., Beilerli A., Ilyasova T., Liang Y., Beylerli O. (2023). The role of circular RNAs in the pathophysiology of oral squamous cell carcinoma. Non-Coding RNA Res..

[7] He S., Chakraborty R., Ranganathan S. (2022). Proliferation and apoptosis pathways and factors in oral squamous cell carcinoma. Int. J. Mol. Sci..

[8] Chang X.-S., Zhu J., Yang T., Gao Y. (2021). MiR-524 suppressed the progression of oral squamous cell carcinoma by suppressing Metadherin and NF-κB signaling pathway in OSCC cell lines. Arch. Oral Biol..

[9] He T., Li X., Xie D., Tian L. (2019). Overexpressed circPVT1 in oral squamous cell carcinoma promotes proliferation by serving as a miRNA sponge. Mol. Med. Rep..

[10] Lindemann A., Takahashi H., Patel A., Osman A.A., Myers J.N. (2018). Targeting the DNA Damage Response in OSCC with TP 53 Mutations. J. Dent. Res..

[11] Naeimi Darestani M., Asl Roosta H., Mosaddad S.A., Yaghoubee S. (2023). The effect of leukocyte- and platelet-rich fibrin on the bone loss and primary stability of implants placed in posterior maxilla: A randomized clinical trial. Int. J. Implant. Dent..

[12] Chiang C.-P., Chang J.Y.-F., Wang Y.-P., Wu Y.-H., Lu S.-Y., Sun A. (2018). Oral lichen planus–differential diagnoses, serum autoantibodies, hematinic deficiencies, and management. J. Formos. Med. Assoc..

[13] Tan Y.-Q., Zhang J., Zhou G. (2023). Autophagy-related 9 homolog B regulates T-cell-mediated immune responses in oral lichen planus. Arch. Oral Biol..

[14] Jaafari-Ashkavandi Z., Fatemi F.-S. (2013). Evaluation of proliferation activity in dysplastic and nondysplastic oral lichen planus through the analysis of argyrophilic nucleolar organizer regions. J. Craniofac. Surg..

[15] Krupaa R.J., Sankari S.L., Masthan K., Rajesh E. (2015). Oral lichen planus: An overview. J. Pharm. Bioallied Sci..

[16] Ranganathan K., Kavitha L. (2019). Oral epithelial dysplasia: Classifications and clinical relevance in risk assessment of oral potentially malignant disorders. J. Oral Maxillofac. Pathol..

[17] Honwad S., Ravi S., Donoghue M., Joshi M. (2017). Immuno-histochemical and quantitative study of melanocytes and melanin granules in oral epithelial dysplasia. J. Clin. Diagn. Res..

[18] Hu T., Weng S., Tang W., Xue R., Chen S., Cai G., Cai Y., Shen X., Zhang S., Dong L. (2015). Overexpression of BIRC6 is a predictor of prognosis for colorectal cancer. PLoS ONE.

[19] Zhuang W., Zhang C., Hao F., Sun X. (2018). Baculoviral IAP repeat containing 6 (BIRC6) is a predictor of prognosis in prostate cancer. Med. Sci. Monit..

[20] Ismail E.A.R., Mahmoud H.M., Tawfik L.M., Habashy D.M., Adly A.A.M., El-Sherif N.H., Abdelwahab M.A. (2012). BIRC6/Apollon gene expression in childhood acute leukemia: Impact on therapeutic response and prognosis. Eur. J. Haematol..

[21] Salehi S., Jafarian A.H., Montazer M., Moghbeli M., Forghanifard M.M. (2017). BRUCE Protein, New Marker for Targeted Therapy of Gastric Carcinoma. J. Gastrointest. Cancer.

[22] Mattila R., Syrjänen S. (2010). Caspase cascade pathways in apoptosis of oral lichen planus. Oral Surg. Oral Med. Oral Pathol. Oral Radiol. Endodontol..

[23] Gómez Bergna S.M., Marchesini A., Amorós Morales L.C., Arrías P.N., Farina H.G., Romanowski V., Gottardo M.F., Pidre M.L. (2022). Exploring the Role of the Inhibitor of Apoptosis BIRC6 in Breast Cancer: A Database Analysis. JCO Clin. Cancer Inform..

[24] Luk I.S.U., Shrestha R., Xue H., Wang Y., Zhang F., Lin D., Haegert A., Wu R., Dong X., Collins C.C. (2017). BIRC6 Targeting as Potential Therapy for Advanced, Enzalutamide-Resistant Prostate CancerBIRC6 as a Target for Enzalutamide-Resistant CRPC. Clin. Cancer Res..

[25] Tang W., Xue R., Weng S., Wu J., Fang Y., Wang Y., Ji L., Hu T., Liu T., Huang X. (2015). BIRC6 promotes hepatocellular carcinogenesis: Interaction of BIRC 6 with p53 facilitating p53 degradation. Int. J. Cancer.

[26] Chomik P., Gil-Kulik P., Filas M., Wojcieszek A., Wilinski M., Karwat J., Kotula L., Niedojadlo A., Czop M., Bogucka-Kocka A. (2014). The expression BIRC6 gene in patients with chronic lymphocytic leukemia—A preliminary study. Curr. Issues Pharm. Med. Sci..

[27] Dong X., Lin D., Low C., Vucic E.A., English J.C., Yee J., Murray N., Lam W.L., Ling V., Lam S. (2013). Elevated expression of BIRC6 protein in non–small-cell lung cancers is associated with cancer recurrence and chemoresistance. J. Thorac. Oncol..

[28] Van Houdt W., Emmink B., Pham T., Piersma S., Verheem A., Vries R., Fratantoni S., Pronk A., Clevers H., Rinkes I.B. (2011). Comparative proteomics of colon cancer stem cells and differentiated tumor cells identifies BIRC6 as a potential therapeutic target. Mol. Cell. Proteom..

[29] Schnoell J., Kadletz L., Jank B.J., Oberndorfer F., Brkic F.F., Gurnhofer E., Cede J., Seemann R., Kenner L., Heiduschka G. (2020). Expression of inhibitors of apoptosis proteins in salivary gland adenoid cystic carcinoma: XIAP is an independent marker of impaired cause-specific survival. Clin. Otolaryngol..

[30] Jaafari-Ashkavandi Z., Aslani E. (2017). Caveolin-1 expression in oral lichen planus, dysplastic lesions and squamous cell carcinoma. Pathol. Res. Pract..

[31] Hao Y., Sekine K., Kawabata A., Nakamura H., Ishioka T., Ohata H., Katayama R., Hashimoto C., Zhang X., Noda T. (2004). Apollon ubiquitinates SMAC and caspase-9, and has an essential cytoprotection function. Nat. Cell Biol..

[32] Gharabaghi M.A. (2018). Diagnostic investigation of BIRC 6 and SIRT 1 protein expression level as potential prognostic biomarkers in patients with non-small cell lung cancer. Clin. Respir. J..

[33] Garrison J.B., Ge C., Che L., Pullum D.A., Peng G., Khan S., Ben-Jonathan N., Wang J., Du C. (2015). Knockdown of the inhibitor of apoptosis BRUCE sensitizes resistant breast cancer cells to chemotherapeutic agents. J. Cancer Sci. Ther..

[34] Chen Y., Fu D., Xi J., Ji Z., Liu T., Ma Y., Zhao Y., Dong L., Wang Q., Shen X. (2012). Expression and clinical significance of UCH37 in human esophageal squamous cell carcinoma. Dig. Dis. Sci..

[35] Banakar M., Ardekani S.T., Zare R., Malekzadeh M., Mirhadi H., Khademi B., Rokaya D. (2022). Oral squamous cell carcinoma: The role of BIRC6 serum level. BioMed Res. Int..

[36] Mallick S., Patil R., Gyanchandani R., Pawar S., Palve V., Kannan S., Pathak K., Choudhary M., Teni T. (2009). Human oral cancers have altered expression of Bcl-2 family members and increased expression of the anti-apoptotic splice variant of Mcl-1. J. Pathol..

[37] Schoelch M., Le Q., Silverman S., McMillan A., Dekker N., Fu K., Ziober B., Regezi J. (1999). Apoptosis-associated proteins and the development of oral squamous cell carcinoma. Oral Oncol..

[38] Poomsawat S., Punyasingh J., Vejchapipat P. (2014). Overexpression of survivin and caspase 3 in oral carcinogenesis. Appl. Immunohistochem. Mol. Morphol..

[39] Li S., Chai L., Cai Z., Jin L., Chen Y., Wu H., Sun Z. (2012). Expression of survivin and caspase 3 in oral squamous cell carcinoma and peritumoral tissue. Asian Pac. J. Cancer Prev..

[40] Low C.G., Luk I.S., Lin D., Fazli L., Yang K., Xu Y., Gleave M., Gout P.W., Wang Y. (2013). BIRC6 protein, an inhibitor of apoptosis: Role in survival of human prostate cancer cells. PLoS ONE.

[41] Dekker N.P., Lozada-Nur F., Lagenaur L.A., MacPhail L.A., Bloom C.Y., Regezi J.A. (1997). Apoptosis-associated markers in oral lichen planus. J. Oral Pathol. Med..

[42] Mosaddad S.A., Mahootchi P., Safari S., Rahimi H., Aghili S.S. (2023). Interactions between systemic diseases and oral microbiota shifts in the aging community: A narrative review. J. Basic Microbiol..

[43] Darestani M.N., Houshmand B., Mosaddad S.A., Talebi M. (2023). Assessing the Surface Modifications of Contaminated Sandblasted and Acid-Etched Implants Through Diode Lasers of Different Wavelengths: An In-Vitro Study. Photobiomodulation Photomed. Laser Surg..

[44] Eslami S., Hosseinzadeh Shakib N., Fooladfar Z., Nasrollahian S., Baghaei S., Mosaddad S.A., Motamedifar M. (2023). The role of periodontitis-associated bacteria in Alzheimer’s disease: A narrative review. J. Basic Microbiol..

[45] Tobón-Arroyave S., Villegas-Acosta F., Ruiz-Restrepo S., Vieco-Durán B., Restrepo-Misas M., Londoño-López M. (2004). Expression of caspase-3 and structural changes associated with apoptotic cell death of keratinocytes in oral lichen planus. Oral Dis..

[46] Leite A.F.S.D.A., Bernardo V.G., Buexm L.A., Fonseca E.C.D., Silva L.E.D., Barroso D.R.C., Lourenço S.D.Q.C. (2016). Immunoexpression of cleaved caspase-3 shows lower apoptotic area indices in lip carcinomas than in intraoral cancer. J. Appl. Oral Sci..

[47] Juneja S., Chaitanya N.B., Agarwal M. (2015). Immunohistochemical expression of Bcl-2 in oral epithelial dysplasia and oral squamous cell carcinoma. Indian J. Cancer.

[48] Ravi D., Nalinakumari K., Rajaram R., Nair M.K., Pillai M.R. (1996). Expression of programmed cell death regulatory p53 and bcl-2 proteins in oral lesions. Cancer Lett..

[49] Javagal V., Rai H. (2015). Inhibitors of Apoptosis: Strong Supporters for Oral Cancer Progression. J. Adv. Med. Dent. Sci. Res..

[50] Muzio L.L., Staibano S., Pannone G., Mignogna M.D., Mariggiò A., Salvatore G., Chieffi P., Tramontano D., De Rosa G., Altieri D.C. (2001). Expression of the apoptosis inhibitor survivin in aggressive squamous cell carcinoma. Exp. Mol. Pathol..

[51] Okazaki Y., Tanaka Y., Tonogi M., Yamane G. (2002). Investigation of environmental factors for diagnosing malignant potential in oral epithelial dysplasia. Oral Oncol..

[52] Hauser H.-P., Bardroff M., Pyrowolakis G., Jentsch S. (1998). A giant ubiquitin-conjugating enzyme related to IAP apoptosis inhibitors. J. Cell Biol..

[53] Yoshihara K., Wang Q., Torres-Garcia W., Zheng S., Vegesna R., Kim H., Verhaak R.G. (2015). The landscape and therapeutic relevance of cancer-associated transcript fusions. Oncogene.

